# Functional Inhibitory Siglec-6 Is Upregulated in Human Colorectal Cancer-Associated Mast Cells

**DOI:** 10.3389/fimmu.2018.02138

**Published:** 2018-09-20

**Authors:** Yingxin Yu, Bart R. J. Blokhuis, Mara A. P. Diks, Ali Keshavarzian, Johan Garssen, Frank A. Redegeld

**Affiliations:** ^1^Division of Pharmacology, Faculty of Science, Utrecht Institute for Pharmaceutical Sciences, Utrecht University, Utrecht, Netherlands; ^2^Division of Digestive Diseases and Nutrition, Department of Internal Medicine, Rush University Medical Center, Chicago, IL, United States; ^3^Nutricia Research, Utrecht, Netherlands

**Keywords:** Siglec-6, human mast cells, immunoreceptor tyrosine-based inhibitory motif, colorectal cancer, hypoxia

## Abstract

Mast cells (MC) accumulate in colorectal cancer (CRC) and the relationship between MC density and cancer progression has been well recognized. MC can be either pro-tumor or anti-tumor players, depending on the local factors present in the tumor microenvironment. Upon malignant transformation, cancer cells express high levels of sialic acids on cell membrane or by secretion. Siglecs are a family of immunoglobulin-like receptors that bind sialic acids and each subtype has a distinct pattern of expression on immune cells. Among them, Siglec-6 is expressed predominately by MC. However, the function of Siglec-6 in MC is largely unexplored and whether it is expressed by CRC-associated MC remains unknown. In this study, we explored the function of Siglec-6 in CD34^+^ derived human MC. MC activation was initiated by IgE crosslinking with or without preincubation of anti-Siglec-6 Ab. Siglec-6 engagement significantly attenuated IgE-dependent MC degranulation as measured by ß-hexosaminidase release and CD63 expression. Interestingly, the production of GM-CSF was also shown reduced upon Siglec-6 engagement. To mimic the milieu of CRC, we cultured primary human MC with colon cancer cells or under hypoxia and Siglec-6 was then measured on these conditioned MC. Coculture with colon cancer cells (HT29 and Caco2) induced upregulation of Siglec-6 on MC. In comparison, normal colon cells (CCD841) had no effect. Also, a time-dependent increase of Siglec-6 by MC was observed under 1% O_2_. Immunohistochemistry of CRC tissue showed expression of Siglec-6 by MC in submucosa. Lectin immunochemistry revealed the presence of actual ligands for Siglec-6 in human CRC tissues. Together, our findings illustrate that Siglec-6 is a functionally inhibitory receptor on MC and suggest that Siglec-6 expression may be relevant for MC activity in the tumor microenvironment of CRC.

## Introduction

MC accumulate in colorectal cancer (CRC) (1–3). It has been well recognized of the relationship between MC density and CRC progression (3–11). MC can be either pro-tumor or anti-tumor players, depending on the local tumor microenvironment, where various immunologic and non-immunologic factors can regulate MC activation and thus influence their role in cancer progression (1, 12, 13). Studies have shown that MC can promote cancer cell survival and proliferation, stimulate angiogenesis, induce immune suppression, but they can also counteract cancer growth by releasing cytotoxic mediators and recruiting immune cells (1, 14–19). Hence, a better knowledge of regulatory signals that control MC activation could help shaping these cells into an anti-tumor player.

Siglecs are a family of immunoglobulin-like receptors that bind sialic acid and predominantly expressed on cells of the immune system (20, 21). In humans, at least 14 Siglecs have been identified and can be divided into two major groups: those that are evolutionally conserved among mammals—such as sialoadhesin (Siglec-1), CD22 (Siglec-2), MAG (Sigelc-4), and Siglec-15—and a group of CD33-related Siglecs that differ in composition across species (20). All Siglecs characteristically possess an N–terminal lectin domain for binding of sialic acid-containing glycan ligands (21). Most of CD33-related Siglecs contain one or more immunoreceptor tyrosine-based inhibition motifs (ITIMs) in their cytoplasmic tail, which transmit inhibitory signals once phosphorylated (21). Since sialic acids are present on all cells, the recognition of self-associated glycans by Siglecs helps to keep resting immune cells in a quiescent state (22, 23). Upon malignant transformation, sialic acids can be overexpressed by cancer cells on membrane glycoproteins and glycolipids or in the manner of secretion (20, 24). These high levels of sialic acids, through the interaction with Siglecs, influence the local immune responses against cancer cells (20, 22, 24–26).

Human MC express Siglec-3, −5, −6, −7, and −8 (21, 27, 28). Among them, Siglec-6 is expressed at the highest level, which has been shown in HMC-1 cell line and CD34^+^ derived human MC (27). However, the functional property of Siglec-6 in MC is largely unexplored and whether it is expressed by CRC-associated MC remains unknown. In this study, we investigated the function of Siglec-6 in CD34^+^ derived human MC by crosslinking with anti-Siglec-6 Ab. Using *in vitro* culture models, the effect of CRC milieu on Siglec-6 expression on primary human MC was analyzed. *In situ* expression of Siglec-6 and its ligands were further analyzed in human CRC tissues.

## Materials and methods

### Ethics statement

Peripheral autologous hematopoietic stem cells derived from patients were used after written informed consent as approved by the ethics committee (TCBio 16-089) of the Utrecht Medical Center, Utrecht, the Netherlands, in accordance with the Declaration of Helsinki (59th WMA General Assembly, Seoul, October 2008), and in compliance with guidelines from the Ethical Committee and European Union legislation. Paraffin sections of human colorectal cancer and healthy colon biopsies were provided by Dr. Ali Keshavarzian from Rush University Medical Center, Chicago, IL, USA. These samples were obtained from the archival paraffin-block tissue in the Department of Pathology after approval for their use from the Institutional Review Board at Rush University Medical Center (ORA #: 13022002-IRB01).

### Generation of primary human MC

CD34^+^ derived human MC were generated from surplus autologous stem cell concentrates as previously described by Schmetzer et al. (29). Briefly, frozen stem cell concentrates were rapidly thawed at 37°C under sterile conditions and poured in a large cell culture flask (Greiner). 20% human serum albumin clinical solution (HSA) (Sanquin), 6% hydroxyethyl starch clinical solution (Braun), and RMPI containing 10 U/ml Heparin (LEO pharma) were then added slowly and consecutively to the cell concentrate. Cells were then filtered through a cell dissociation sieve (Sigma) and incubated with DNAse (200 U.I./ml, Roche) for 15 min. After washed, cells were re-suspended in PBS containing 4% HSA and incubated with Fc-Block (Miltenyi) for 15 min, CD34^+^ positive selection cocktail (StemCell) for 15 min and nanoparticles for 10 min. Subsequently, CD34^+^ cells were sorted with an EasySep® Magnet (StemCell) according to the manufacturer's protocol. Finally, sorted cells were re-suspended in serum-free expansion medium (SFEM) (StemCell) supplemented with human LDL (50 μg/ml, StemCell). On day 1, human recombinant IL-3 (100 ng/ml, Biolegend), and SCF (100 ng/ml, Miltenyi) were added. Every three to four days, IL-3 and SCF were added to a final concertation of 20 ng/ml. At the end of the second week, MC were maintained under 20 ng/ml SCF with the withdrawal of IL-3. From day 17 till day 28, cells were used in described experiments. The subset of mature MC was identified by flow cytometry based on the gating strategy of dead cell exclusion followed by double positive selection of CD117 (eBioscience) and FcεRIa (eBioscience) using BD FACSCanto II.

### MC activation assay

Primary human MC were primed with human IgE (1 μg/ml, Merck) overnight at 37°C. Cells were then incubated with or without mouse anti Siglec-6 mAb (R&D, MAB2859) or mouse isotype IgG (R&D) at 5 μg/ml for 1 h on ice. After washed, cells were subsequently challenged with a range of concentrations (0–4 μg/ml) of rabbit anti-human IgE (Dako) and anti-mouse IgG (Fab')2 (5 μg/ml, Jackson ImmunoResearch) for 90 min or 16 h at 37°C. For beta-hexosaminidase (β-hex) assay, cell-free supernatants were collected after 90 min and incubated with 200 μM 4-methylumbelliferyl-β-d-glucopyranoside (4-MUG) for 1 h at 37 °C. Enzyme reaction was then terminated by adding 0.1 M glycine buffer. As positive control, cells were lysed with 5 % Triton X-100 in order to quantify the total β-hex content. The β-hex content was quantified by measuring fluorescence at ex360/em460 nm. The percentage of β-hex release was calculated as: (A–B)/(T–B) × 100%, where A is the amount of β-hex released from stimulated cells, B is that released from unstimulated cells, and T is total β-hex content released from positive control. For quantification of IL-8 and GM-CSF, cell-free supernatants were harvested after 16 h and analyzed using ELISA kits of human IL-8 (Invitrogen) and GM-CSF (R&D) according to the manufacturer's instruction. Net production of cytokines was calculated as: the amount of IgE-stimulated cytokines subtracts the spontaneous release from unstimulated cells.

### Colon cell lines and coculture

Human colon cancer cell lines HT29 and Caco2, and human normal colon cell line CCD841 were obtained from American Type Tissue Culture Collection. HT29 were grown in McCoy medium (Gibco) supplemented with heat-inactivated 10% fetal calf serum (FCS), and 100 μg/ml pen/strep (penicillin and streptomycin, Gibco, 26600080). Caco2 and CCD841 were cultured in EMEM medium (Lonza, BE12-611F/12) supplemented with 10% FCS, 100 μg/ml pen/strep, 1% non-essential amino acids, 1% L-glutamine and 1% sodium pyruvate (Gibco, 11360-039). All cells were cultured in a humidified 37°C/5% CO_2_ incubator. Prior to coculture 1 × 10^5^ human colon cells were seeded in a 24-well plate. Standard culture medium were removed after 24 h and cells were washed with PBS. Thereafter 1 × 10^6^ primary human MC resuspended in SFEM supplemented with SCF were added to the colon monolayer. Cells were harvested after 72 h of coculture and stained with an apoptotic cell dye YO-PRO1 and antibodies of FcεRIa and CD117. The subset of CD117^+^ FcεRIa^+^ cells was gated by flow cytometry and analyzed for expression of Siglec-6 (R&D, FAB2859P).

### Immunoblotting

Purified human MC were used to obtain protein lysates. Briefly, cells were purified using a dead cell removal kit (Miltenyi) followed by CD117 positive selection (Miltenyi) according to the manufacturer's protocol. The cell viability and purity were assessed by flow cytometry based on the surface expression of an apoptotic cell stain YO-PRO1, antigen CD117 and FcεRIa (Supplemental Figure 1). 1 × 10^6^ purified human MC were then cocultured with or without colon cell lines and subsequently harvested and lysed for 30 min on ice with RIPA buffer (ThermoFisher) supplemented with protease and phosphatase inhibitors (Roche). Samples were then boiled for 5 min in SDS sample loading buffer (62.5 mM Tris-HCL (pH 6.8), 2% sodium dodecyl sulfate (SDS), 0.01% bromophenol blue, 10% glycerol, 100 mM Dithiothreitol), and loaded onto a 4–20% gradient Tris-glycine SDS polyacrylamide gel (Bio-Rad). Proteins were then transferred to a polyvinylidene difluoride membrane (Bio-Rad), and nonspecific binding was blocked with 5% milk in TBST (TBS/0.1% Tween 20) for 1 h. The blot was then washed with TBST and incubated overnight at 4°C with rabbit anti-human Siglec-6 antibody (Sigma, HPA009084) in 5% BSA in TBST. Subsequently, the blot was washed with TBST and incubated for 1 h at room temperature with HRP goat-anti rabbit antibody (DAKO) in 5% milk in TBST. Immunoreacted bands were visualized using ECL prime (Amersham). Proteins were quantified with Image Lab software. The expression of Siglec-6 was normalized to human tryptase levels.

### RNA sequencing

Purified MC were isolated using a BD Influx™ cell sorter to obtain RNA samples. Briefly, primary human MC were stained with an apoptotic cell dye YO-PRO1 and antibodies for CD117 and FcεRIa. The subset of YO-PRO1^−^ CD117^+^ FcεRIa^+^ cells was sorted (Supplemental Figure 2). RNA of purified MC was isolated using PureLink RNA mini kit (ThermoFisher) according to the manufacturer's protocol. RNA samples were then sequenced on an Illumina Nextseq500 platform according to the manufacture's procedure by the Utrecht Sequencing Facility of the Utrecht University (http://www.useq.nl/). Sequencing libraries were generated using TruSeq Stranded mRNA poly A kit. Sequence reads were checked for quality by FastQC (v0.11.4) after which reads were aligned to GRCh37 using STAR (v2.4.2a) and read groups were added using Picard (v1.141). All samples passed QC and were subsequently processed using HTSeq-count (v0.6.1) on ENSEMBL gene definitions (GRCh37, release 74). RPKM (Reads Per Kilobase of transcript, per Million mapped reads) was calculated to quantify gene expression.

### Immunohistochemistry

Siglec-6 expression on paraffin-embedded tissue sections was assessed by immunofluorescence histochemistry. Paraffin-embedded tissue sections were deparaffinized with xylene and graded ethanol. Antigen retrieval was performed by heating the sections in DAKO target retrieval solution (Dako) for 10 min in a microwave, with intermediate cooling incubations of 30 min. Tissue sections were then blocked with 10% goat serum for 1 h at room temperature. Sections were then incubated with mouse anti-human tryptase (Abcam) or mouse IgG isotype and rabbit anti-human Siglec-6 (Sigma, HPA009084) or rabbit IgG isotype for overnight at 4°C and were subsequently detected with Alexa Fluor 488 goat anti-mouse IgG (ThermoFisher) and Alexa Fluor 594 goat anti-rabbit IgG (Invitrogen). Nuclei were counterstained with DAPI (Invitrogen). Tissue sections were analyzed by BZ9000 fluorescence microscopy (Keyence) and TCS-SP8 confocal microscopy (Leica).

Siglec-6 ligand expression on paraffin-embedded tissue sections was assessed by lectin immunohistochemistry. Paraffin-embedded tissue sections were treated in the same manner as previously mentioned. Endogenous peroxidase activity was blocked with 3% H_2_O_2_ for 1 h at room temperature. Specific staining was done with 5 μg/ml biotinylated Siglec-6 Fc chimera (R&D, 2859-SL) for overnight at 4°C, or with biotinylated mouse anti-rat IgG as negative control. Biotinylated Siglec-6 Fc chimera was generated using Mix-n-Stain biotin labeling kit (Biotium). Avidin-conjugated horseradish peroxidase (HRP) was used as detection reagent (VECTASTAIN Elite ABC kit). The complex was visualized with chromogen substrate DAB. All tissue sections were counterstained with hematoxylin. Tissue sections were analyzed by Olympus BX50 microscopy (Leica).

### Statistical analysis

Results are expressed as mean ± SEM or mean ± SD. Data were analyzed by Student's *t*-test, one-way or two-way ANOVA followed by *post-hoc* Dunnett's or Sidak's multiple comparison test. The analyses were performed with the GraphPad Prism statistical program. *P*-values < 0.05 were deemed as significant.

## Results

### Primary human MC constitutively express siglec-6

**Figure 1:**
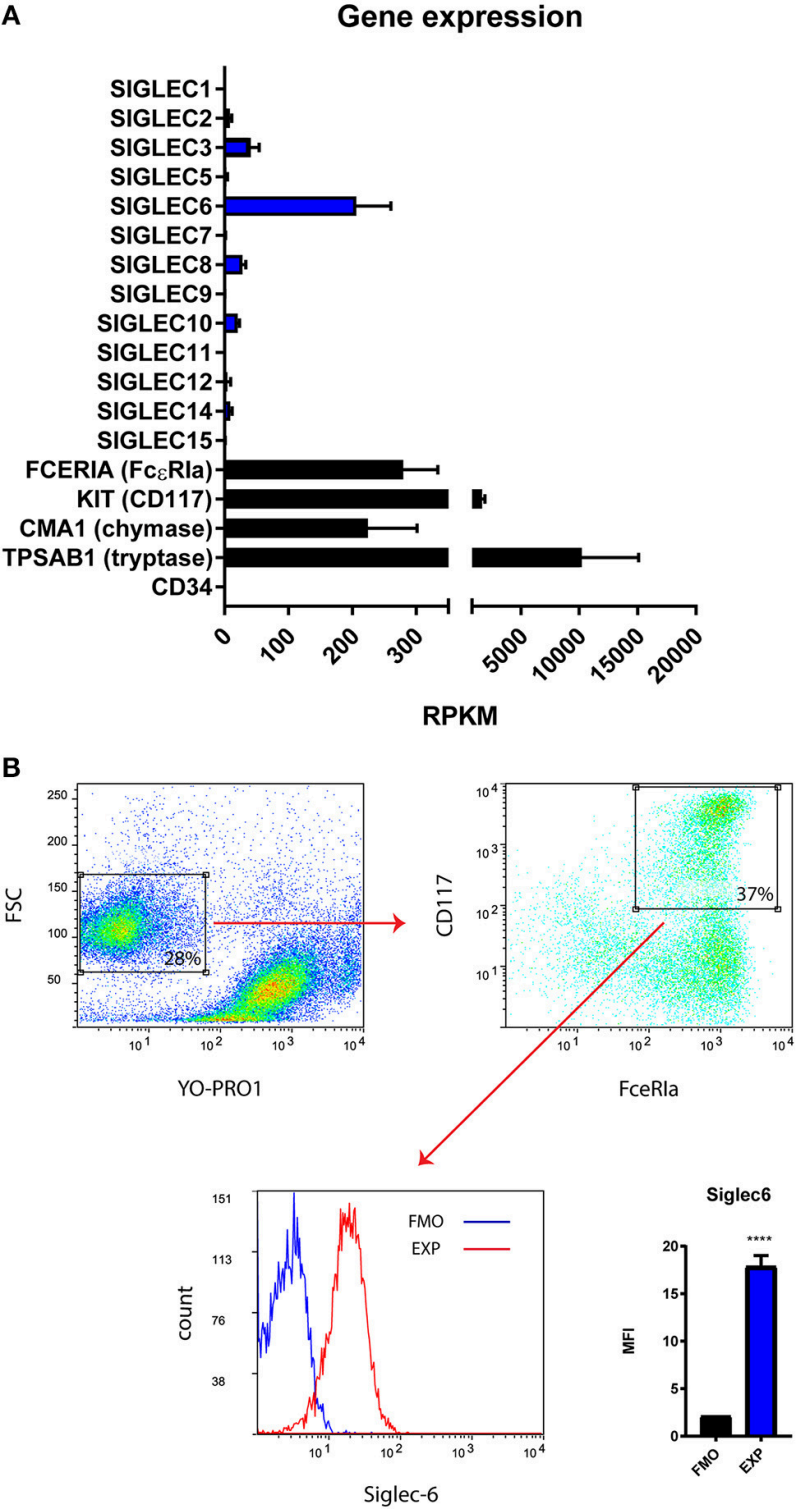
Primary human MC constitutively express Siglec-6. **(A)** Gene expression (Reads Per Kilobase of transcript, per Million mapped reads, RPKM) of multiple SIGLECs in purified primary human MC (*n* = 2). CD34 was negative control and MC marker genes (FCERIA, KIT, CMA1, TPSAB1) were positive control for the *in vitro* maturation of human MC. **(B)** Gating strategy to identify the subset of MC based on dead cell exclusion followed by double positive selection of CD117 and FcεRIa. The purified subset of MC was selected for Siglec-6 analysis on the cell surface. Median fluorescence intensity (MFI) for fluorescence minus one control (FMO) and Siglec-6 staining (EXP) was compared. ^****^P < 0.0001, Student's *t*-test. Data are representative of 3 independent experiments.

Primary human MC were cultured from CD34^+^ autologous stem cell preparations. To determine the extent to which Siglecs are expressed by human MC, we isolated the purified MC subset from primary cell cultures, using FACs cell sorter (Supplemental Figure 2) and subsequently performed RNA sequence analysis. Negative expression of CD34 and positive expression of KIT (CD117), FCERIA (FcεRIa), CMA1 (chymase) and TPSAB1 (tryptase) indicated maturation of these human MC (Figure 1A). Multiple SIGLEC genes (SIGLEC-2, −3, −6, −8, −10, −12, and −14) were detected and expressed at various levels in human MC (Figure 1A). Among them, SIGLEC6 was found to be highly expressed (Figure 1A). To confirm the protein level of SIGLEC-6 in human MC, the purified subset of MC was gated (YO-PRO1^−^ CD117^+^ FcεRIa^+^) and analyzed by flow cytometry (Figure 1B). As expected, high levels of Siglec-6 were detected on the cell surface of human MC (Figure 1B).

### Siglec-6 engagement inhibits IgE-mediated MC activation

**Figure 2:**
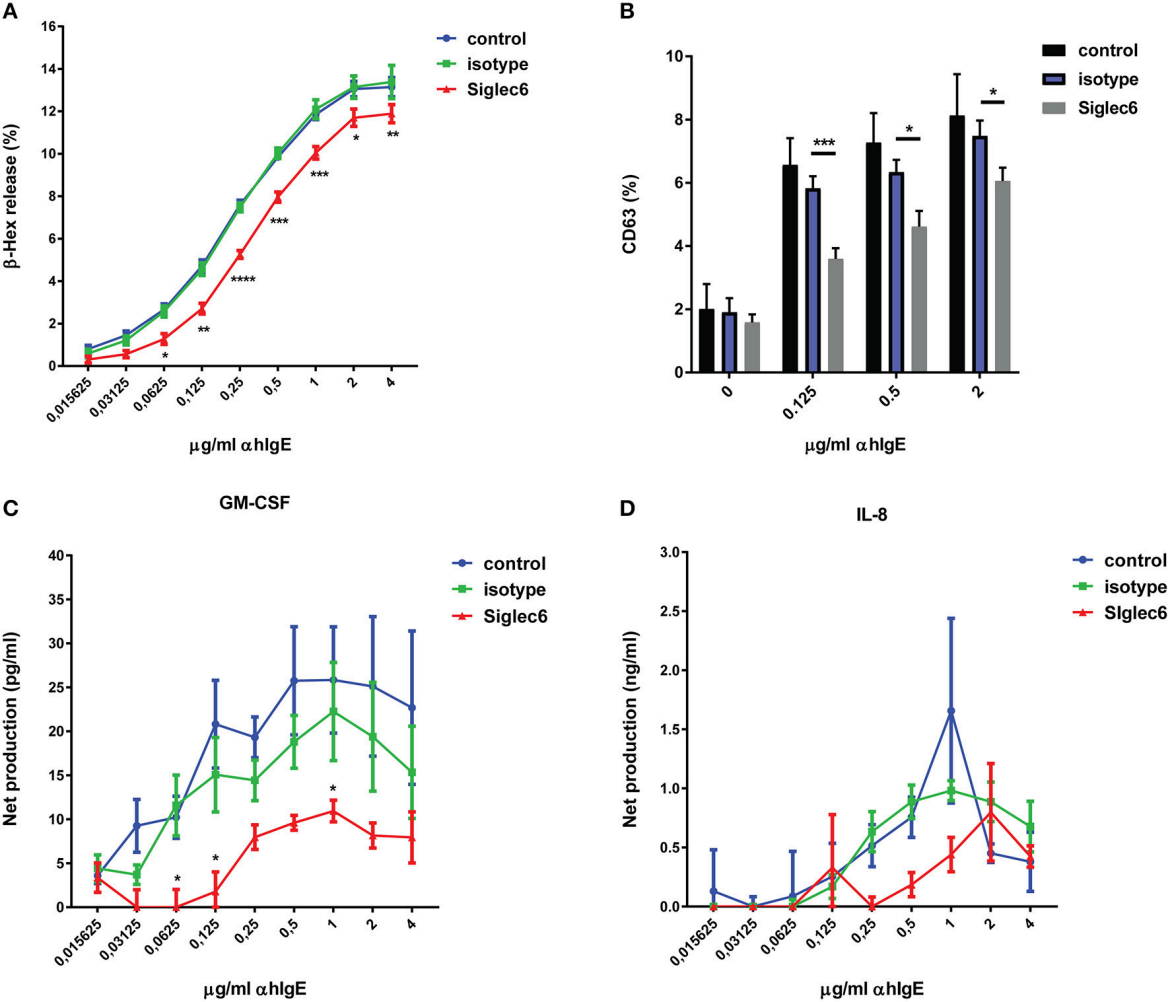
Siglec-6 engagement inhibits IgE-mediated human MC activation. Primary human MC were primed with human IgE overnight, and then incubated in the absence or presence of mouse IgG (isotype) or mouse anti-human Siglec-6 (Siglec6). Subsequently, cells were exposed to control medium or various concentrations of anti-human IgE (α hIgE). Supernatants were collected after 90 min to measure beta-hexosaminidase (β-hex) **(A**). The purified subset of MC was selected for analysis of CD63 expression **(B)**. Supernatants were collected after 16 h to measure GM-CSF **(C)** and IL-8 **(D)**. ^*^P < 0.05; ^**^P < 0.005; ^***^P < 0.001; ^****^P < 0.0001, two-way ANOVA. Values are mean ± SEM of 3 independent experiments.

Based on the presence of ITIMs in Siglec-6 (30), we next investigated if engagement of Siglec-6 inhibits MC activation. Mouse anti-Siglec-6 mAb was used as an artificial ligand to specifically engage Siglec-6. Primary human MC were sensitized with human IgE and subsequently crosslinked with anti-human IgE Ab (α hIgE) to stimulate degranulation. As shown in Figure 2A, α hIgE concentrations ranging from 0.03 to 4 μg/ml induced dose-response of beta- hexosaminidase (ß-hex) release. When MC were treated with isotype control, there was no effect (Figure 2A). In comparison, preincubation with anti-Siglec-6 resulted in ~30% inhibition of ß-hex release (*P* < 0.05, Figure 2A). Furthermore, these inhibitory effects was not due to cell apoptosis caused by Siglec-6 engagement (Supplemental Figure 3). Consistent with ß-hex release, a similar reduction caused by Siglec-6 was observed in CD63 levels, which is an activation marker on MC membrane (*P* < 0.05, Figure 2B).

To further explore the scope of inhibition induced by Siglec-6 engagement, we investigated the effect of Siglec-6 on IgE-triggered newly generated cytokine release from human MC. Cells were treated in the same setting as Figure 2A and supernatants were harvested after 16 h for measuring GM-CSF and IL-8. IgE crosslinking stimulated maximal production of 32.54 ± 14.49 pg/ml GM-CSF per 2 × 10^5^ cells (unstimulated control: 6.8 ± 2.94 pg/ml) and 2.5 ± 1.53 ng/ml IL-8 per 2 × 10^5^ cells (unstimulated control: 0.84 ± 0.08 ng/ml) (data not shown). Of note, Siglec-6 engagement caused 50% or greater reduction of GM-CSF release, compared with isotype control (*P* < 0.05, Figure 2C). However, no significant inhibition of IL-8 was observed, although a slight decrease was seen over the range of α hIgE from 0.25 to 1 μg/ml (Figure 2D).

### Siglec-6 is upregulated on primary human MC in coculture with colon cancer cells or under hypoxia

**Figure 3:**
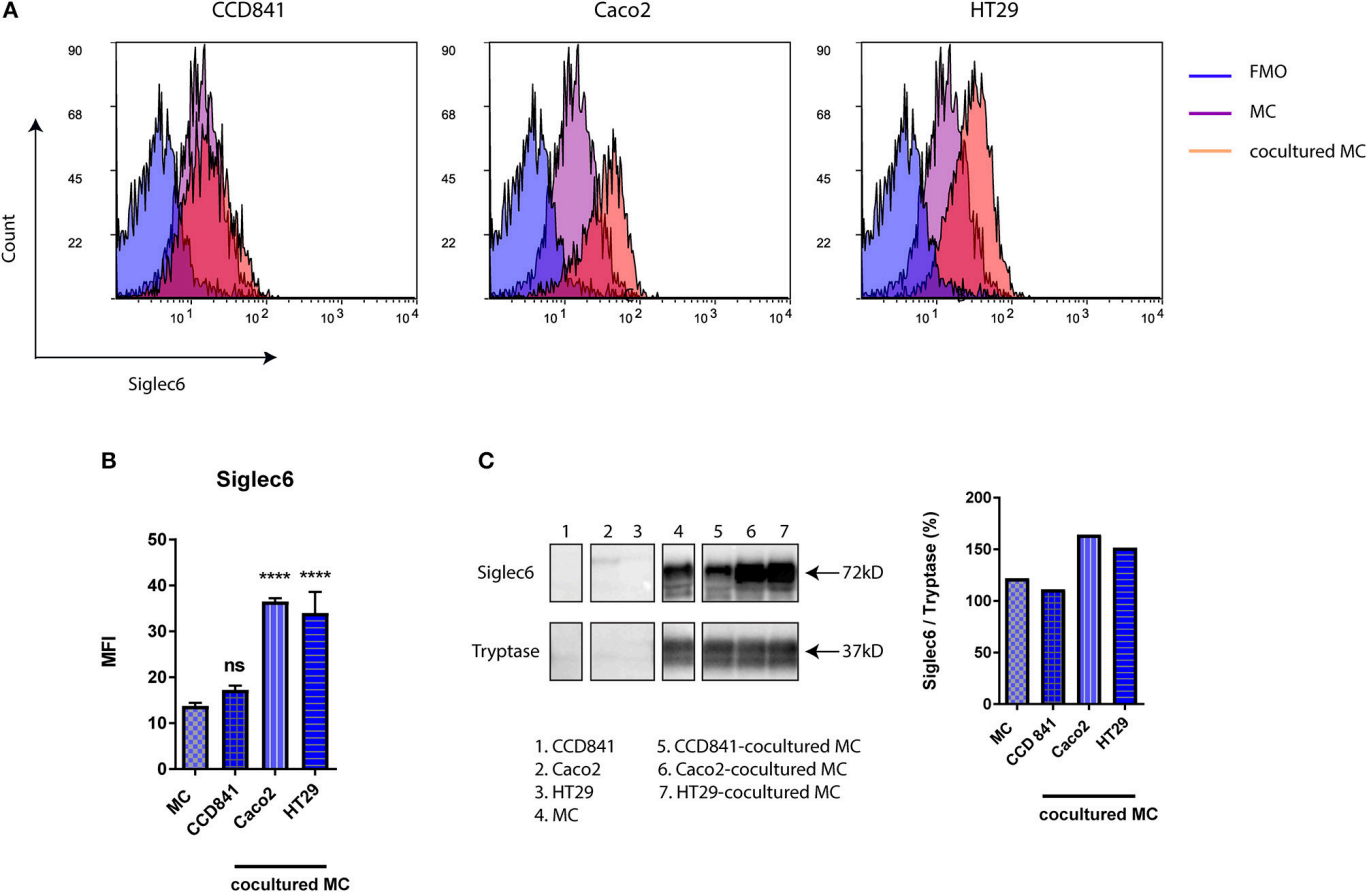
Siglec-6 is upregulated on colon cancer-cocultured MC. **(A)** Representative histograms of surface Siglec-6 on primary human MC cocultured with or without colon cell lines (CCD841, Caco2, and HT29). **(B)** MFI of surface Siglec-6 was compared on primary human MC cocultured with vs. without colon cell lines. **(C)** Immunoblotting of Siglec-6 in colon cell lines and primary human MC cocultured with or without colon cell lines. Tryptase was used as an internal control and quantification of Siglec-6 was normalized to tryptase density. ^****^P < 0.0001, one-way ANOVA. Data are representative of 2–5 independent experiments.

By expressing high levels of sialic acids on the cell surface or on secreted molecules, cancer cells interact with Siglecs on immune cells and thereby modify local immune responses (21, 25, 31). MC are one of earliest recruited immune cells in colorectal cancer (CRC) and their role in CRC has been well recognized (1, 2, 14, 18). To determine if Siglec-6 expression could be modified by colon cancer cells, primary human MC were cocultured with colon cancer cell lines (HT29 and Caco2) or a normal colon cell line (CCD841). After 72 h, cells were harvested and the subset of MC was gated by flow cytometry and further analyzed for Siglec-6 expression. Interestingly, colon cancer cells induced a significant increase of Siglec-6 on MC surface (*P* < 0.0001), while normal colon cells had no effect (Figures 3A,B). To exclude a possibility that increased Siglec-6 could be simply due to its translocation to the cell surface, purified MC was cocultured with colon cell lines and then harvested for immunoblotting of Siglec-6 in whole cell lysates. Similarly, upregulation of Siglec-6 was observed in colon cancer-cocultured MC (Figure 3C and Supplemental Figure 4). In addition, no Siglec-6 expression was detected in colon cell lines (Figure 3C and Supplemental Figure 4), indicating the upregulation of Siglec-6 was caused by the production by MC themselves.

**Figure 4:**
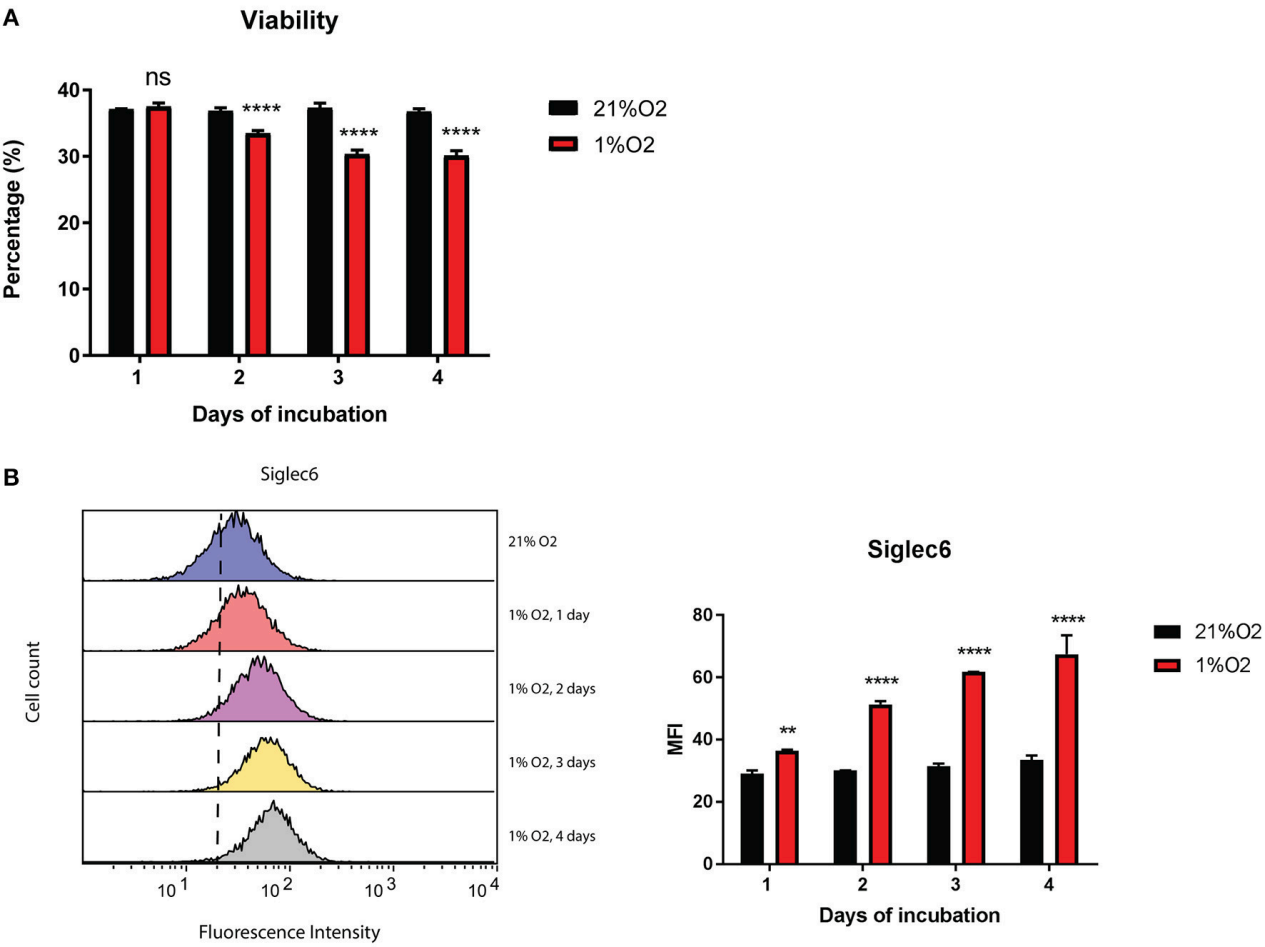
Siglec-6 is upregulated on hypoxia-incubated MC. **(A)** Viability of primary human MC incubated at 21% O2 or 1% O2 for 1–4 days. Only the viable subset of MC was gated for Siglec-6 analysis by flow cytometry. **(B)** Left graph: representative histograms of surface Siglec-6 on primary human MC incubated at 21% O2 or 1%O2 for 1 to 4 days; right graph: MFI of surface Siglec-6 was compared on primary human MC incubated at 21% O2 vs. 1%O2 for 1 to 4 days, respectively. ^**^P < 0.01; ^****^P < 0.0001, two-way ANOVA. Data are representative of at least 2 independent experiments.

Hypoxia is a well-known feature of cancer and thus may influence the phenotype of immune cells during cancer progression (1). Therefore, we examined the effect of low oxygen (1% O2) on Siglec-6 expression in human MC. Although viability of primary human MC dropped significantly since 2nd day of incubation at 1% O2, it decreased only 17% even when cells were in hypoxia for 4 days (Figure 4A). Only the viable subset of MC was gated for further Siglec-6 expression by flow cytometry. Intriguingly, there was a time-dependent increase in membrane Siglec-6 in response to hypoxia and incubation at 1% O2 for 1 day was sufficient to induce a significant upregulation of Siglec-6 on MC membrane (*P* < 0.05, Figure 4B).

### *In situ* expression of siglec-6 by MC in human colorectal cancer

**Figure 5:**
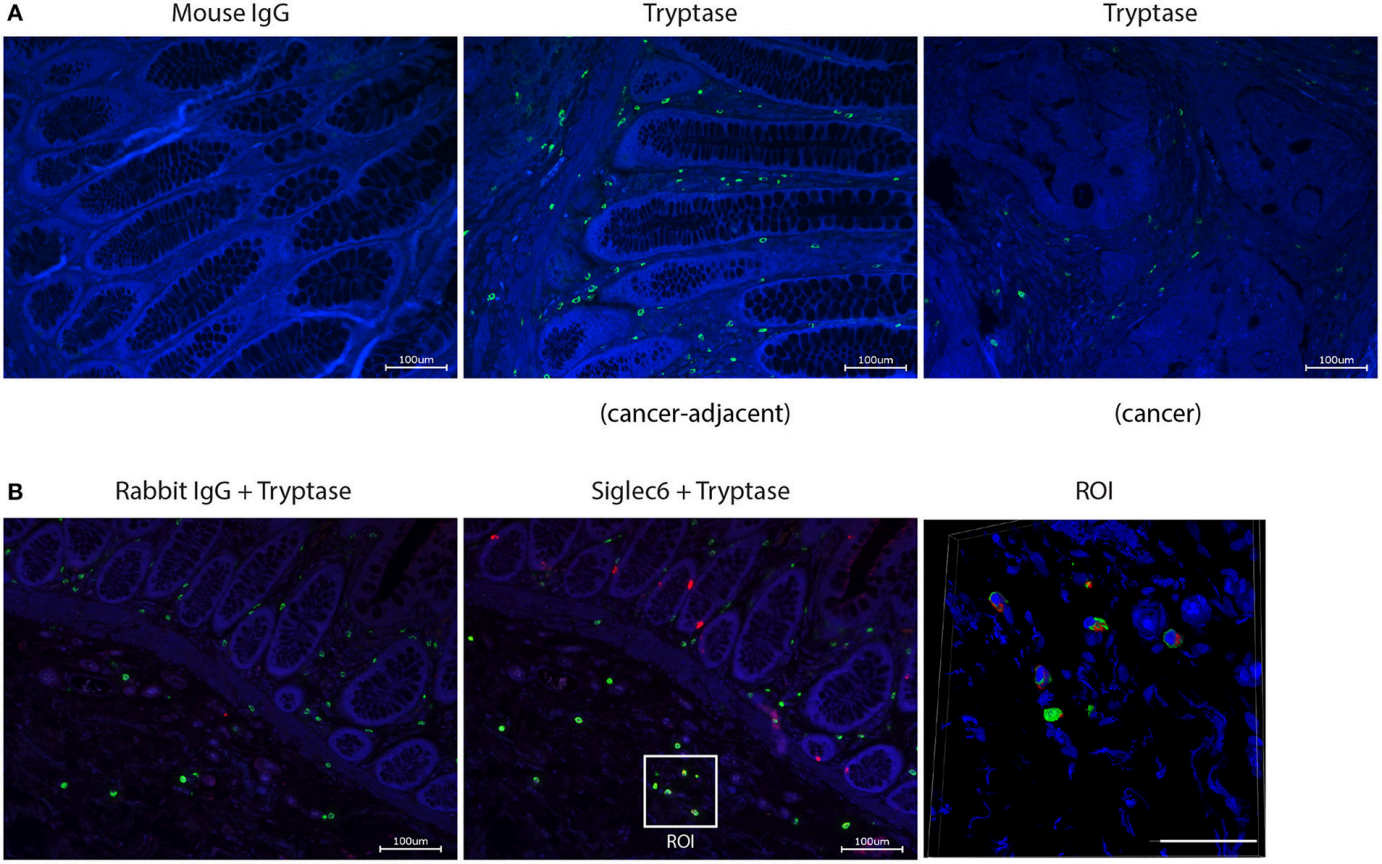
*In situ* expression of Siglec-6 by MC in human CRC tissues. **(A)** Tryptase^+^ MC in CRC stromal and adjacent tissues (cell nuclei: blue; tryptase: green). Mouse IgG isotype was isotype control for mouse anti-human tryptase. **(B)** Siglec-6^+^ MC in CRC-adjacent submucosa (cell nuclei: blue; tryptase: green; Siglec-6: red). Rabbit IgG was isotype control for rabbit anti-human Siglec-6. Region of interest (ROI) was further analyzed by confocal microscopy and Siglec-6^+^ MC was displayed in a merged image from multiple Z stacks (Scale bar, 50 μm). Data are representative of 12 samples.

We next investigated whether Siglec-6 is expressed by CRC-localized MC. To confirm *in situ* expression of Siglec-6, double immunofluorescence staining of tryptase and Siglec-6 was performed on paraffin-embedded sections of human CRC tissues (*n* = 12). Numerous tryptase-positive MC were found in CRC stromal and cancer-adjacent tissues (Figure 5A). Surprisingly, Siglec-6 positive MC were mainly found in cancer-adjacent submucosa (Figure 5B and Supplemental Figure 5). In contrast, mucosal MC did not show Siglec-6 expression, which was also confirmed in healthy colon tissues (*n* = 15) (Supplemental Figure 6). Double positive staining of Siglec-6 and tryptase was further analyzed by confocal microscopy (Figure 5B). 3D images of multiple Z stacks revealed a surface expression pattern of Siglec-6 on CRC submucosal MC (Supplemental Figure 4 and Supplemental Video). In addition, expression of Siglec-6 was also observed in intestinal glands (Figure 5B and Supplemental Figure 5).

### Presence of siglec-6 ligands in human colorectal cancer

**Figure 6:**
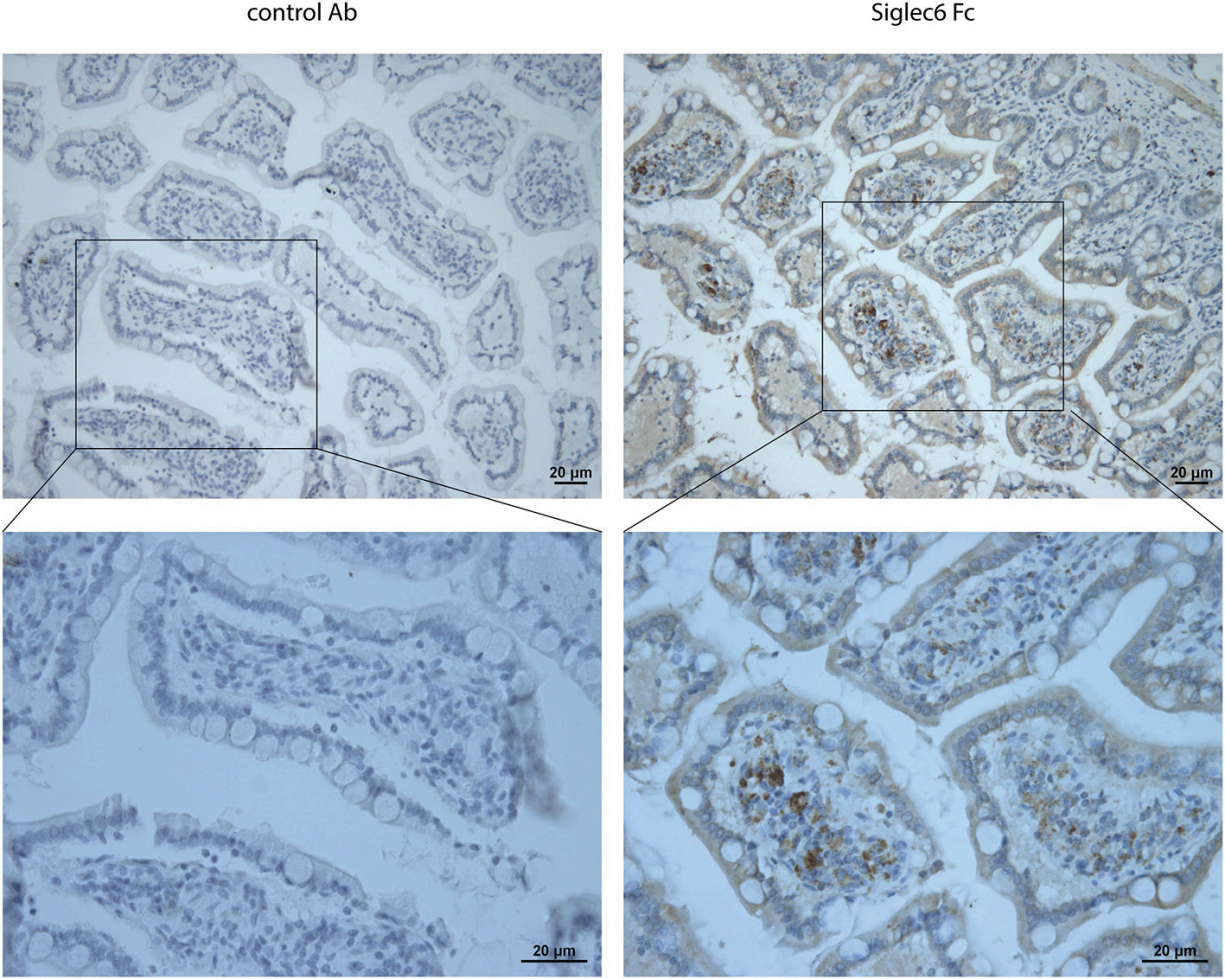
Ligands for Siglec-6 in human CRC tissues. Lectin immunohistochemistry for Siglec-6 ligands in human CRC tissues using Siglec-6-Fc chimera. Mouse IgG was negative control. Data are representative of 5 samples.

Whereas sialic acid is ubiquitously expressed in cancer, only very specific, distinct sialoglycan structures are recognized by individual Siglec receptors (21, 32). Thus, we next investigated if actual ligands for Siglec-6 are present in CRC. To probe for such ligands, we analyzed the binding of a recombinant soluble Fc chimeric protein of Siglec-6 (Siglec-6-Fc) to paraffin-embedded sections of human CRC tissues (*n* = 5). Accumulation of Siglec-6 ligands was found in 3 out of 5 patients and was mainly located in cancerous lesion (Figure 6). In contrast, non-cancerous tissues did not show an accumulation of ligands but only scattered expression (Supplemental Figure 7).

## Discussion

In this study, we presented for the first time *in situ* expression of Siglec-6 by MC and actual ligands for Siglec-6 in human CRC tissues. Functionally, Siglec-6 is an inhibitory receptor on human MC, which upon engagement induced reduction of IgE-dependent ß-hex release and GM-CSF production. Interestingly, it was upregulated on primary human MC in response to colon cancer cells or hypoxia, determining factors in the milieu of CRC. Together, our data suggest that Siglec-6 expression may be relevant for MC activity in the tumor microenvironment of CRC.

In comparison to other primary human MC culture procedures using cord blood or buffy coat as the source for progenitor cells (33–35), the employed culture method from autologous stem cell concentrates resulted in rapid differentiation of CD34^+^ cell into functional MC as of day 17 of culture (29). Due to the thawing of frozen stem cell concentrates, this procedure resulted in a large amount of dead cells in the culture. Other limitation of this culture is the low percentage of CD117 present on MC membrane, due to the internalization of CD117 during the *in vitro* culture, as described by Schmetzer et al. (29). Nevertheless, all the analyses in the present study were done on the purified population of YO-PRO1^−^ CD117^+^ FcεRIa^+^. Interestingly, after 2 weeks of culture, Siglec-6 was already detected on the membrane of these primary MC. This is consistent with the previous study of Yokoi et al., where they showed human MC generated from buffy coat acquire Siglec-6 since the 2nd week of culture (27). Notably, in addition to its early appearance, Siglec-6 was the highest in gene expression among other Siglecs in these primary human MC. Besides, Siglec-6 has been reported to be selectively expressed by MC among other immune cells (21). Together, its features exhibit a potential to be an effective MC-specific target.

To study the functional activity a specific mAb was used as artificial ligand, as has been done earlier for other Siglecs (21, 36). Upon engagement, Siglec-6 attenuated IgE-mediated MC degranulation. Furthermore, no co-crosslinking of Siglec-6 with FcεRI was needed for its inhibitory function. This is analogous to Siglec-8, which upon engagement inhibits FcεRIa-dependent MC degranulation without the requirement of co-crosslinking (37), but is different from Siglec-7, where simultaneous co-crosslinking with FcεRI is indispensable (28). Besides, CD63 expression was also measured in parallel with ß-hex release, which further confirmed the inhibitory effect of Siglec-6 on the single cell level. Intriguingly, different from Siglec-8 (37), engagement of Siglec-6 inhibited the production of GM-CSF by IgE-stimulated MC. No significant reduction in IL-8 production was observed, which may be caused by the high variation in IL-8 production among different human MC batches. Siglec-6 shares a similar molecular structure with other CD33-related members, which contains a cytoplasmic tail with a membrane-proximal ITIM and a membrane-distal ITIM-like domain (21). Previous data of Siglec-3, −7, and −9 showed that recruitment of tyrosine phosphatases, such as SHP-1 and SHP-2, is responsible for the inhibitory effect (38–40). Thus, it could be speculated that SHP recruitment might be involved in Siglec-6 signaling pathways. Of note, contrasting results of Siglec-7 signaling were found in different cells: whereas SHP recruitment is pronounced in Siglec-7 transfected RBL cells (39), it is not the main transduction molecule responsible in primary human MC or NK cells (28, 40). Therefore, further work is needed to elucidate the downstream signaling involved in Siglec-6-mediated inhibition in primary human MC.

Siglecs exhibit different specificities for glycan ligands (21, 30). For instance, Siglec-8 specifically recognizes 6′ sulphated sialyl lewis X (41). Siglec-6 preferentially binds to α2-6-linked sialosides, which is found on placenta and uterine epithelium (42). It has been shown that Siglec-Fc chimeras are powerful tools for detecting specific types and arrangements of sialic acids *in situ* (22, 25). Indeed, our study shows the presence of actual ligands for Siglec-6 in human CRC tissues, indicating a possible interaction of sialoglycans and Siglec-6 in the tumor microenvironment of CRC. Thus, it could be speculated that colon cancer cells might express sialoglycans and thus attenuate MC activation through the interaction with Siglec-6. However, when human MC were cocultured with colon cancer cells and simultaneously stimulated by IgE-crosslinking, no difference was observed between MC cocultured with and without colon cancer cells (Supplemental Figure 8), but it is unclear if Siglec-6 activation occurred by natural ligands in *in vitro* co-culture. It has been shown that expression levels of CD33-related Siglecs are upregulated in tumor-infiltrating T cells in different human carcinomas (43) and the upregulation of Siglec-14 on monocyte has been observed in SLE patients (44). In this study, we found that Siglec-6 was upregulated on primary human MC when cultured with colon cancer cells or stimulated by hypoxia. This upregulation was not simply caused by cell coculture, as no effect was observed in the coculture with a normal colon cell line. On the other hand, this effect might not be limited to colon cancer cells or Siglec-6. Common features of malignant transformed cells and hypoxia-treated cells might be responsible. A previous study of Yin et al has shown that hypoxia culture induced acquisition of N-glycolyl sialic acid on colon cancer cells (45). It is reasonable to speculate that human MC may acquire particular sialic acids under hypoxia and thereby stimulating the increased surface levels of Siglecs. Interestingly, we showed expression of Siglec-6 by MC in human CRC tissue. *In situ* expression of Siglec-6 in CRC-associated MC was limited to cancer-adjacent submucosa, and negative in cancer stromal and adjacent mucosal tissue. In agreement with our study, no expression of Siglec-6 was found in lung mucosal MC (21, 27). Although mucosal MC in healthy colorectal tissue were negative, no submucosal MC were present in the stained tissue specimen and yet no conclusion can be made on the extent of expression of Siglec6 in CRC vs. healthy colon tissue. Our future studies will further focus which local factors may be involved in inducing the expression of Siglec-6 and if increased Siglec6 expression leads to enhanced inhibition in the CRC milieu.

In conclusion, although Siglec-6 was originally identified as a placenta protein (20, 21), in the immune system, it is a surface lectin distinctively expressed by MC and weakly on basophils and B cells (20, 21, 46). To date, cell-directed therapies are increasingly of therapeutic interest (20), and a role of Siglecs in improving the innate immune response to cancer is emerging (22, 25, 26). In this study, we showed for the first time the *in situ* expression of Siglec-6 by MC in human CRC tissues. Our data illustrated that Siglec-6 is a functionally inhibitory receptor on human MC and that this receptor is upregulated on MC when stimulated with colon cancer cells or hypoxia. These findings support a concept that MC activity may be modulated through Siglec-6 in the tumor microenvironment of CRC.

## Author contributions

YY and FR designed the experiments. YY, BB, and MD performed experimental procedures. YY performed data collection, analysis and drafted the manuscript. JG and FR supervised the program. AK provided paraffin-embedded sections of human colorectal cancer tissues and health colon tissues. All authors listed have made significant contribution to writing the manuscript and approved it for publication.

### Conflict of interest statement

The authors declare that the research was conducted in the absence of any commercial or financial relationships that could be construed as a potential conflict of interest.
