# Supplementary material for: Functional Inhibitory Siglec-6 Is Upregulated in Human Colorectal Cancer-Associated Mast Cells
Source: Front Immunol. 2018 Sep 20;9:2138. doi: 10.3389/fimmu.2018.02138 (PMC6159741; doi:10.3389/fimmu.2018.02138)
Supplement: Supplementary file 2 [file Data_Sheet_1.pdf]

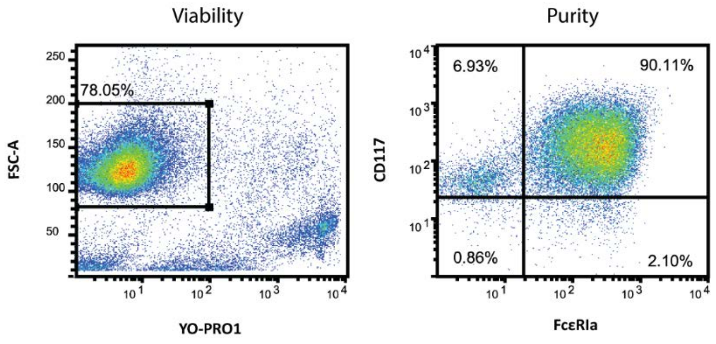

S Figure 1. Viability and Purity of purified human MC

Left graph: cells with negative staining of a apoptotic cell dye YO-PRO1 were identified as viable. Right graph: cells with double positive expression of CD117 and FcεRIa were identified as MC.

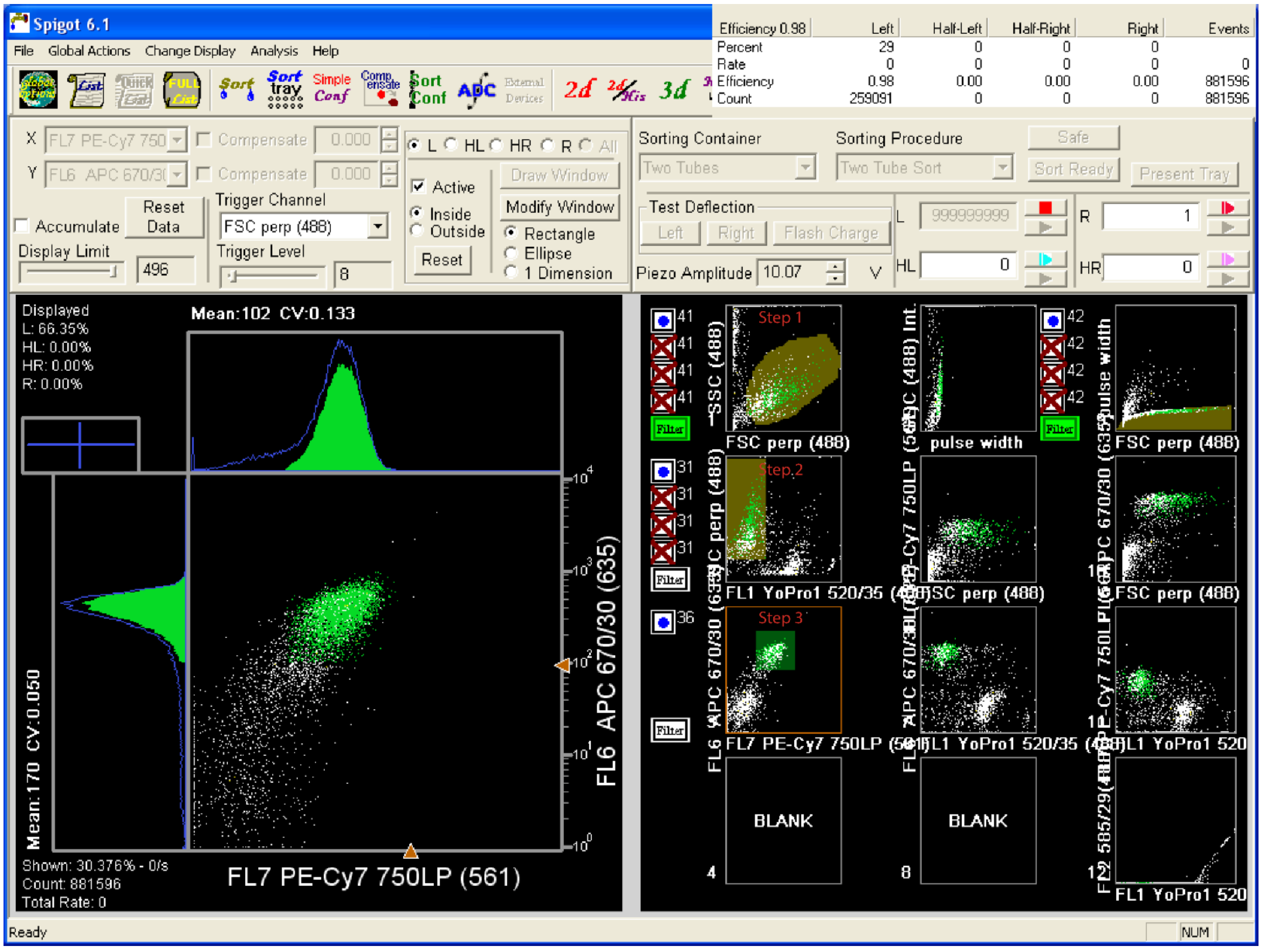

S Figure 2. The sorting strategy of purified MC subsets

Cells were stained with YO-PRO1 dye, CD117 and FcεRIa antibodies. (Right window) Step 1: to exclude cell debris, cells were selected based on FSC and SSC; Step 2: viable cells were selected based on negative YO-PRO1 staining; Step 3: mature MC were then selected based on double positive staining of APC-labeled CD117 and PE-Cy7-labeled FcεRIa. (Left window) Cells were sorted from consecutive three gating steps (green area).

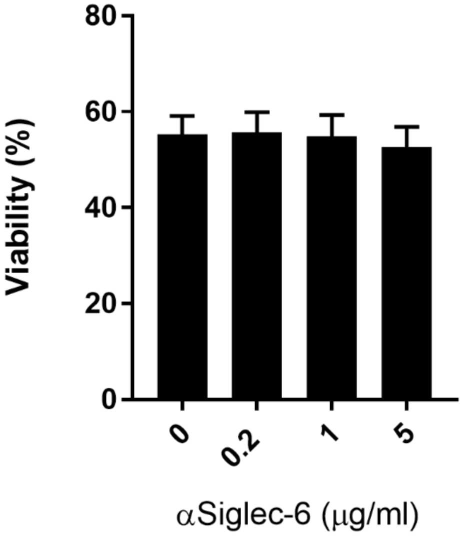

S Figure 3. Viability of MC in response to anti-Siglec-6 mAb

Cells were treated in the same manner as Fig.2 and subsequently harvested for measurement of viability by flow cytometry based on YO-PRO1 staining. Data are representative of 3 independent experiments.

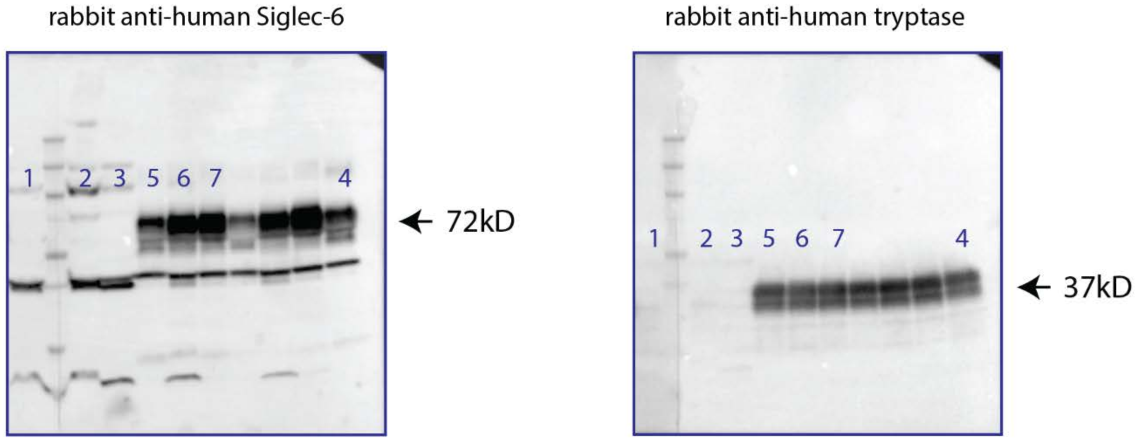

S Figure 4. Full scan of the entire original immunoblot for Figure 3C.

1. CCD841
2. Caco2
3. HT29
4. HMC
5. HMC + CCD841
6. HMC + Caco2
7. HMC + HT29

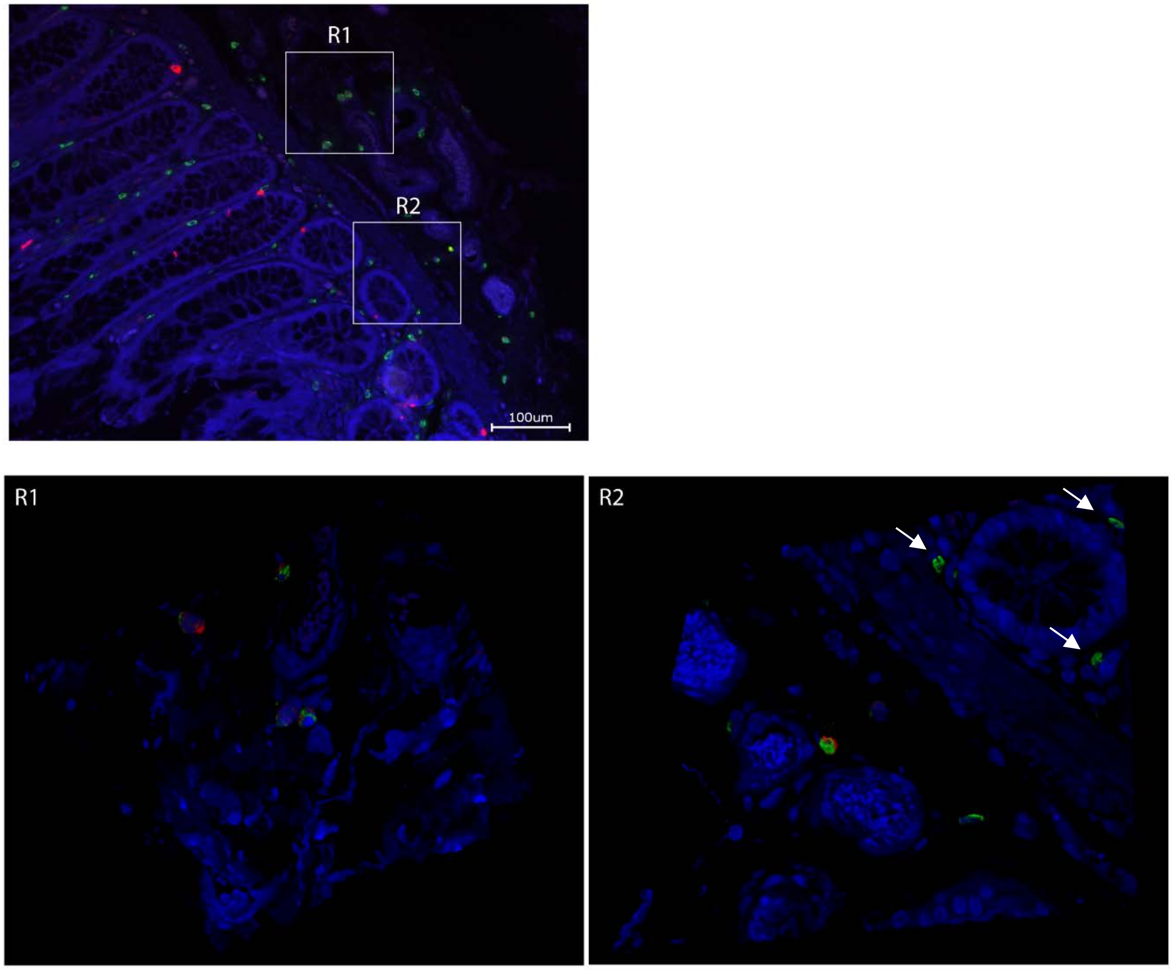

S Figure 5. In situ expression of Siglec-6 in CRC-adjacent submucosal MC

Siglec-6$^+$ MC were found in CRC-adjacent submucosa (cell nuclei: blue; tryptase: green; Siglec-6: red). Regions of interest (R1 and R2) were further analyzed by confocal microscopy. Merged images of multiple Z stacks are shown for Siglec-6$^+$ MC and no siglec-6 staining was observed in mucosal MC (arrows, R2) (original magnification, 600 ×). Data are representative of 12 independent experiments.

Supplemental video. Siglec-6 expression by CRC-adjacent submucosal MC

Cell nuclei are shown in blue, tryptase positive mast cells are shown in green and Siglec-6 staining in red (original magnification, 600 ×).

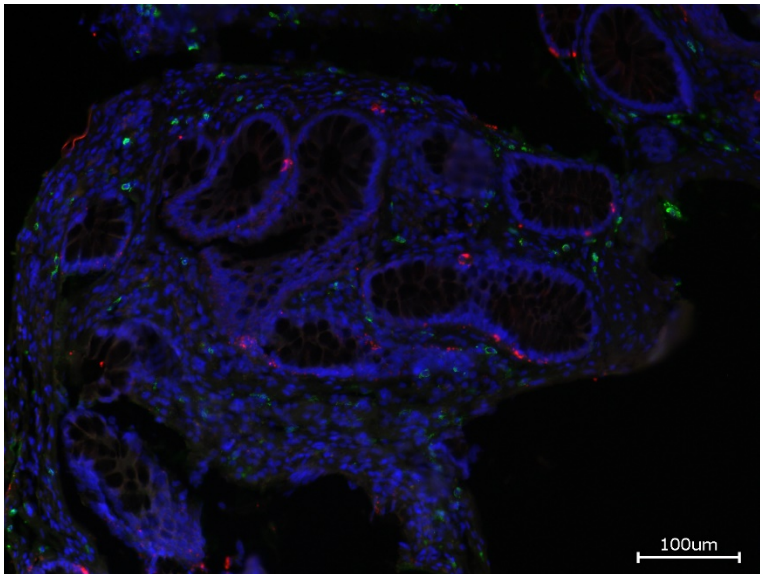

S Figure 6. Mucosal MC in healthy colon do not show Siglec-6 expression

Cell nuclei are shown in blue, tryptase in green and Siglec-6 in red. Data are representative of 15 independent experiments. Samples are 8 µm deep cut sections from human healthy colon.

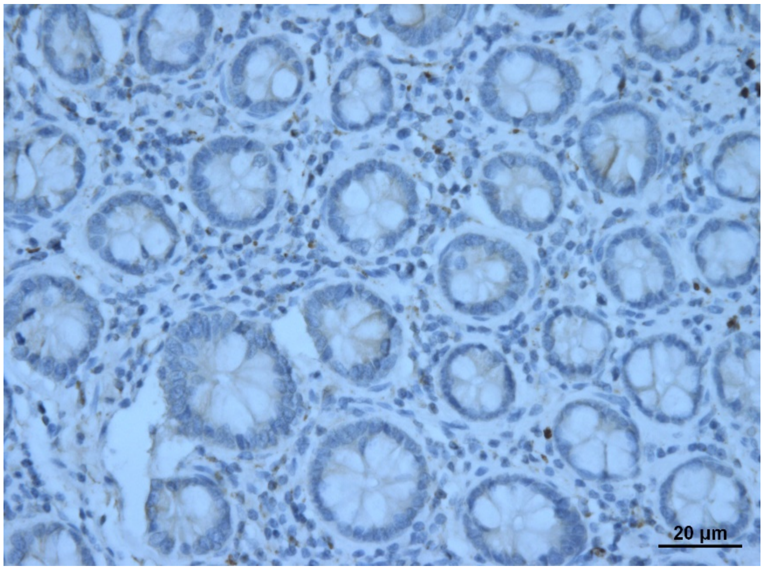

S Figure 7. Expression of Siglec-6 ligands in CRC-adjacent mucosa

Lectin immunohistochemistry for Siglec-6 ligand expression using Siglec-6-Fc chimera in human CRC-adjacent mucosa. Data are representative of 5 independent experiments.

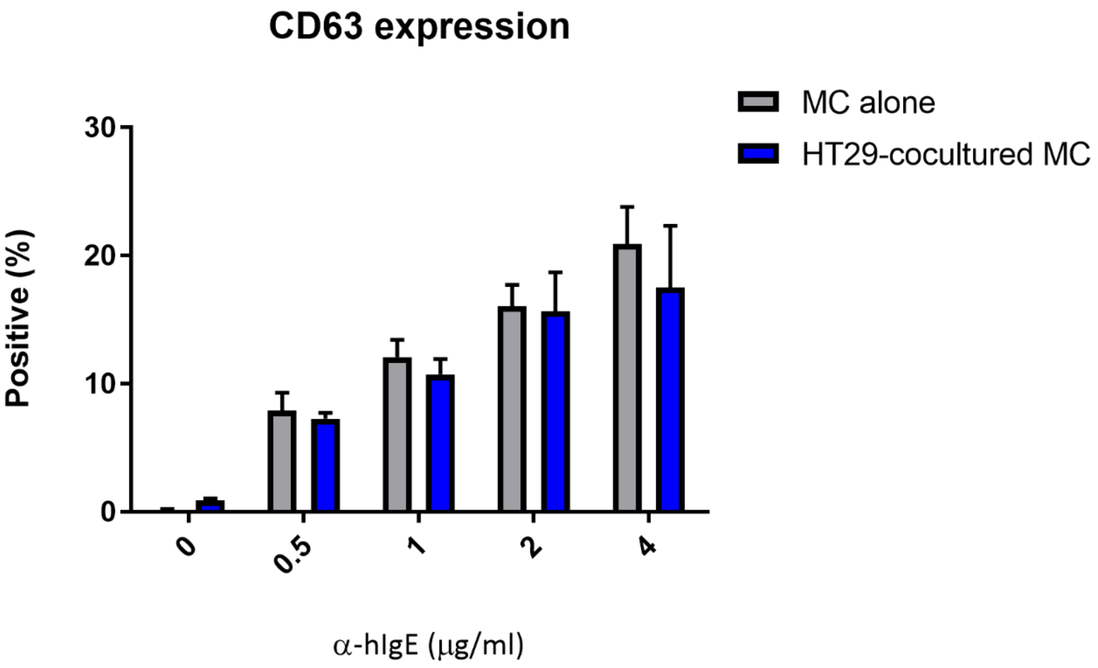

S Figure 8. Comparison of IgE-mediated activation from MC cocultured with and without HT29

Human MC were cocultured with or without HT29 for 72 hours and directly stimulated with IgE-crosslinking in the coculture. After 90 min, cells were harvested and a population of MC was gated and analyzed for CD63 expression. Values are mean ± SEM of 3 independent experiments.
